# Intracellular Dual Behavior of Trolox in HeLa Cells and 3T3 Fibroblasts Under Basal and H_2_O_2_-Induced Oxidative Stress Conditions

**DOI:** 10.3390/molecules30183755

**Published:** 2025-09-16

**Authors:** Maria Elena Giordano, Maria Giulia Lionetto

**Affiliations:** 1 Department of Biological and Environmental Sciences and Technologies, University of Salento, 73100 Lecce, Italy; 2 NBFC, National Biodiversity Future Center, 90100 Palermo, Italy

**Keywords:** Trolox, antioxidant activity, prooxidant effect, oxidative stress, HeLa cells, 3T3 fibroblasts, CM-H_2_DCFDA

## Abstract

Trolox, a water-soluble analog of vitamin E, is widely used as a reference antioxidant in in vitro biochemical assays. However, its intracellular redox behavior is known to vary depending on both concentration and oxidative context. In this study, we investigated the dose-dependent antioxidant and prooxidant effects of Trolox in two cellular models, HeLa cells and 3T3 cells exposed for 1 h to increasing concentrations (2–160 µM), under both basal conditions and oxidative stress induced by hydrogen peroxide. Intracellular oxidative changes were assessed using the oxidative stress-sensitive fluorescent probe CM-H_2_DCFDA. Under basal conditions, Trolox exerted slight dose-dependent antioxidant behavior in 3T3 cells on the basal production of ROS in concentrations ranging from 2 µM to 160 µM. In contrast, in HeLa cells Trolox displayed a biphasic activity: antioxidant at low doses (≤10 µM) and a switch to prooxidant behavior at higher concentrations. Under H_2_O_2_-induced stress, in HeLa cells Trolox retained antioxidant activity at low concentrations (≤10 µM), but this effect gradually declined at higher doses, disappearing around 80 µM and shifting to a slight prooxidant effect at 160 µM. Confocal microscopy confirmed the spectrofluorimetric results. Conversely, 3T3 cells exhibited an early shift toward prooxidant activity already at 10 µM. These findings highlight that the Trolox redox activity is determined not only by concentration but also by cell-specific intracellular environment and redox state. The study suggests caution against generalized antioxidant use of Trolox and highlights the need for specific dose–response evaluations in specific cell types and biological settings.

## 1. Introduction

**Figure 1:**
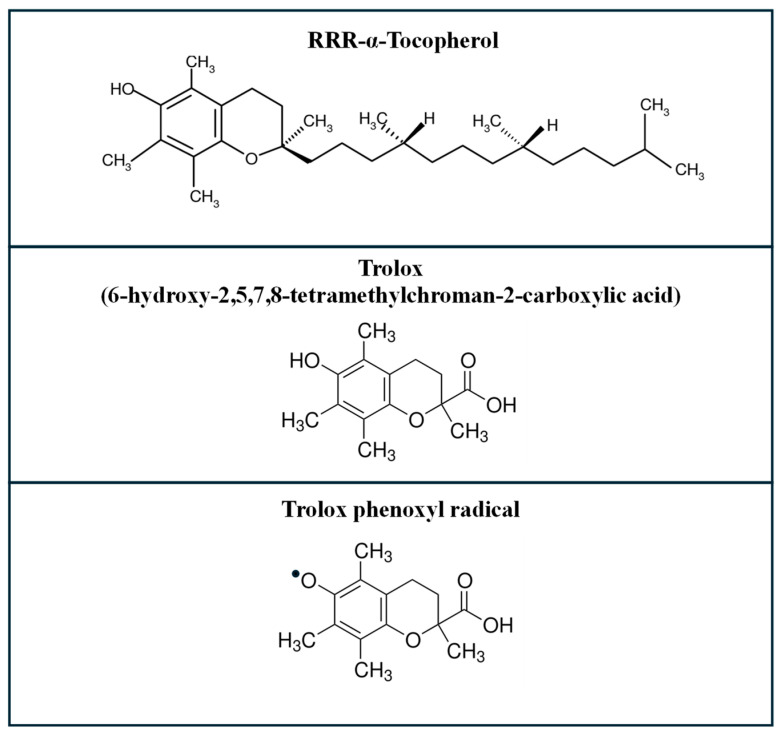
Chemical structure of α-tocopherol, Trolox, and Trolox phenoxyl radical.

Trolox (6-hydroxy-2,5,7,8-tetramethylchroman-2-carboxylic acid) is a water-soluble analog of vitamin E (α-tocopherol), the most biologically active and abundant form of tocopherol in humans. Figure 1 illustrates the structural formula of both Trolox and α-tocopherol, with RRR-α-tocopherol representing the natural stereoisomer of α-tocopherol. Trolox has a stereogenic center at C-2 that can give rise to two enantiomers, (2R) and (2S); however, it is commercially available predominantly as a racemic mixture.

While α-tocopherol is a major lipid-soluble antioxidant in biological membranes, neutralizing lipid peroxyl radicals and inhibiting peroxidation of polyunsaturated fatty acids [1,2], Trolox retains similar antioxidant functions but lacks the hydrophobic phytyl tail, making it significantly more water-soluble [3]

This enhanced solubility facilitates its use in aqueous biological systems, where α-tocopherol is less effective, and has led to its widespread use as a reference antioxidant in various a-cellular assays (e.g., ABTS, ORAC, FRAP) [4,5,6,7]. In such assays, Trolox is a potent scavenger of hydroxyl, alkoxy, and peroxyl radicals, operating through hydrogen atom transfer, radical adduct formation, single-electron transfer, and sequential proton loss electron transfer mechanisms [8,9].

In lipid environments, however, its reactivity is more limited, primarily relying on hydrogen atom transfer and adduct formation mechanisms. Radical scavenging of species like ˙OCH_3_, ˙OOH, and ˙OOCHCH_2_ has been shown to occur predominantly via hydrogen atom transfer at physiological pH, shifting toward sequential proton loss electron transfer at alkaline pH [8]. Upon oxidation, Trolox forms a relatively stable phenoxyl radical (Figure 1), similar to that of α-tocopherol, which can be recycled by reducing agents like ascorbate or glutathione [9].

Trolox has demonstrated a range of beneficial cellular effects, often surpassing α-tocopherol in protective efficacy in various cell types, including human myocytes, hepatocytes, and erythrocytes [10,11,12]. It has also been shown to reduce oxidative stress-induced apoptosis in diabetic models [13]. Moreover, it reduced oxidative stress, neuroinflammation, and motor impairment in mice with Parkinson’s-like symptoms [14]. In combination with coenzyme Q10, Trolox protected retinal cells from glutamate-induced damage, restored mitochondrial function, and maintained vascular health in the eye [15].

Despite its widely recognized antioxidant properties, Trolox can also exhibit prooxidant activity under specific conditions, particularly in the presence of transition metals or when cellular antioxidant systems are depleted. In such environments, Trolox may participate in redox cycling and metal chelation, promoting ROS generation rather than scavenging [16,17,18]. For instance, it can potentiate oxidative stress induced by arsenic trioxide (As_2_O_3_) in malignant cells, while sparing non-malignant cells [18].

Our previous study [3] demonstrated for the first time that Trolox’s antioxidant/prooxidant behavior is concentration-dependent in HeLa cells exposed to Trolox for 24 h under baseline (no added oxidant) conditions and related to apoptotic progression. This study highlighted that dose plays a critical role in switching the activity of Trolox from protective to potentially harmful, yet this concentration-dependent behavior remains poorly characterized in the literature, especially under oxidative stress conditions.

In the present study, we build upon our previous findings by examining the intracellular antioxidant and prooxidant effects of Trolox in two cellular models, HeLa cells and 3T3 cells, exposed to oxidative stress induced by hydrogen peroxide (H_2_O_2_). To further refine our understanding of its dose-dependent behavior, we employed a shorter pre-treatment window (1 h) with Trolox, followed by 1 h exposure to H_2_O_2_, simulating acute oxidative insult.

Hydrogen peroxide was selected due to its role as a physiological prooxidant, since it is naturally produced by enzymes such as NADPH oxidases [19] and through the mitochondrial electron transport chain [20]. At regulated concentrations under the control of the cellular antioxidant defense systems, H_2_O_2_ is vital for normal cell signaling and metabolic processes [19,21]. However, when its levels become unregulated, rising above the cellular antioxidant capacity, it can lead to oxidative stress and cellular damage. Additionally, H_2_O_2_ is a major reactive oxygen species generated during inflammation (by neutrophils and macrophages) [22], with its concentration varying by tissue type, physiological context, and the severity of the inflammatory response. Generally, H_2_O_2_ concentration ranges from low micromolar to sub-millimolar levels under inflammatory conditions [23,24,25]. Specifically, concentrations ranging from 100 to 500 µM have been detected locally in inflamed tissues, including arthritic synovial fluid [26], solid tumors [27], and ischemic regions [28].

For this study, we selected 3T3 fibroblasts and HeLa cells as complementary models to investigate the antioxidant and prooxidant activity of Trolox. 3T3 cells, derived from non-transformed mouse fibroblasts, represent a healthy, low-ROS environment ideal for assessing Trolox’s antioxidant effects under physiological conditions [29].

In contrast, HeLa cells, originating from human cervical cancer, are known for their high basal oxidative stress [30]. Moreover, HeLa cells have also been recently employed to evaluate the intracellular antioxidant and prooxidant effects of Trolox under the baseline condition (no added oxidant) in our previous study [3]. The use of HeLa cells enables direct comparison of short-term exposure under the prooxidant condition in this study, with results previously obtained under the baseline condition during 24 h exposure.

The approach in this study based on two cell types enables a comparative analysis of Trolox’s redox behavior in normal versus cancer-like cellular contexts. By extending the investigation to stress conditions and shorter exposure times, this study wants to provide new insights into the dynamic antioxidant/prooxidant role of Trolox within a biologically relevant timeframe and context.

To our knowledge, this is the first study to systematically and comparatively evaluate the concentration-dependent switch in Trolox activity under acute oxidative stress conditions using a short-term exposure model. By integrating two complementary cell lines and physiologically relevant H_2_O_2_ concentrations, this work aims to advance current knowledge on Trolox’s dual redox behavior in a time- and context-dependent manner.

## 2. Results

### 2.1. Effect of TROLOX on Intracellular Basal ROS Production in Hela Cells and 3T3 Cells After 1 h Incubation

It is known that 3T3 fibroblasts represent a low-ROS cellular model, whereas HeLa cells are characterized by high basal oxidative stress [29,30]. Preliminarily, we confirmed this basal difference between the two cell models by comparing their basal fluorescence intensity values. Both cell types were seeded at the same density (1 × 10^4^ cells/mL) in a Corning^®^ (New York, NY, USA) 96-well black plate, incubated for 24 h to allow adhesion, then loaded with the ROS-sensitive probe CM-H_2_DCFDA, and their basal fluorescence levels were measured. Figure S1 shows the basal fluorescence of HeLa cells expressed as a percentage of that in 3T3 cells. Basal fluorescence in HeLa cells was approximately 170% of that measured in 3T3 cells.

**Figure 2:**
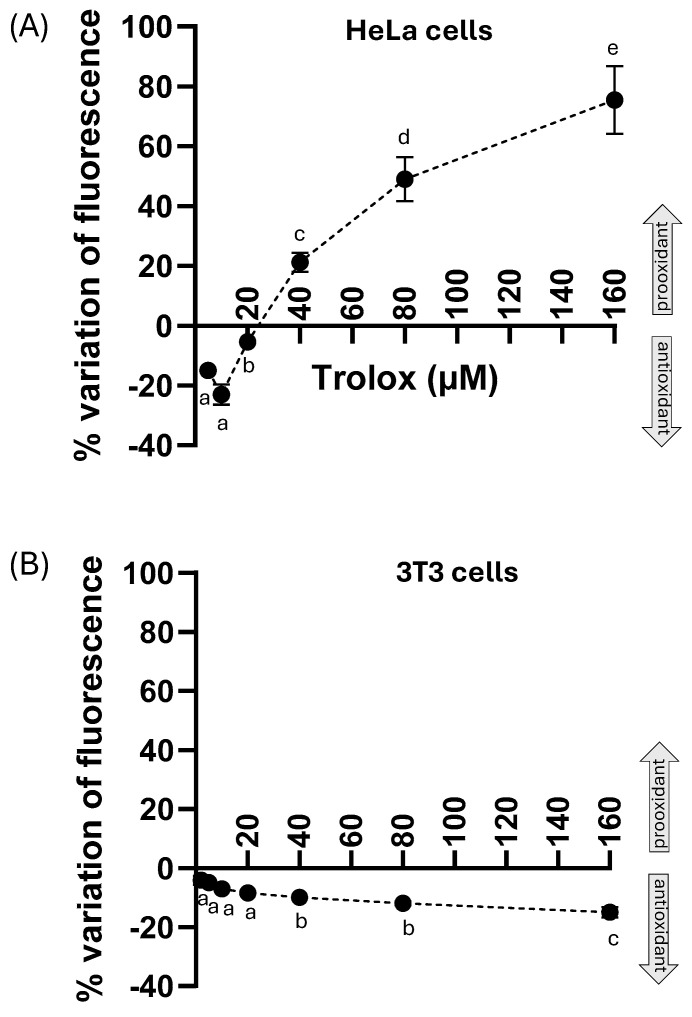
Effect of Trolox (1 h incubation) at different concentrations (from 2 µM to 160 µM) on the endogenous ROS in HeLa cells (**A**) and 3T3 cells (**B**). The *y*-axis represents the percentage variation of the fluorescence intensity in the cells exposed for 1 h to different concentrations of Trolox (2–160 µM), followed by loading with the ROS-sensitive probe 5-(and-6)-chloromethyl-2′,7′-dichlorodihydrofluorescein diacetate (CM-H_2_DCFDA) for 30 min. The percentage variation of fluorescence was calculated using the formula (fluorescence of treated cells—fluorescence of control cells)/fluorescence of control cells × 100. Data are presented as the mean ± SEM from three independent experiments. The statistical significance of data was analyzed by one-way ANOVA followed by Tukey’s multiple comparison post-test. Different letters indicate statistically significant differences (*p* < 0.05) between concentrations.

To assess the effect of TROLOX on intracellular basal ROS production in Hela cells and 3T3 cells under short-term exposure conditions, both HeLa cells and 3T3 cells were treated for 1 h with increasing concentrations of Trolox (2–160 µM) and subsequently loaded with the cell-permeable, ROS-sensitive probe CM-H_2_DCFDA. Figure 2A,B show the percentage variation in fluorescence relative to controls, represented by cells not treated with Trolox. The percentage variation with respect to the control was calculated according to the formula (treated − control)/control × 100. In HeLa cells (Figure 2A), at lower concentrations (2–10 µM), Trolox reduced basal ROS levels, as indicated by the negative percentage change in CM-H_2_DCFDA fluorescence, reflecting an antioxidant effect of approximately 20% at a concentration of 10 µM. However, this antioxidant effect diminished at 20 µM and was no longer detectable at higher concentrations. In contrast, at concentrations ranging from 40 to 160 µM, Trolox exhibited a dose-dependent prooxidant effect, evidenced by a positive increase in the fluorescence percentage variation. This result demonstrates a dose-dependent dual behavior of Trolox on the basal ROS production of HeLa cells following 1 h exposure. In 3T3 cells (Figure 2B), Trolox exerted a slight dose-dependent antioxidant activity on the basal production of ROS in concentrations ranging from 2 µM to 160 µM.

### 2.2. H_2_O_2_-Induced Intracellular Prooxidant Conditions

**Figure 3:**
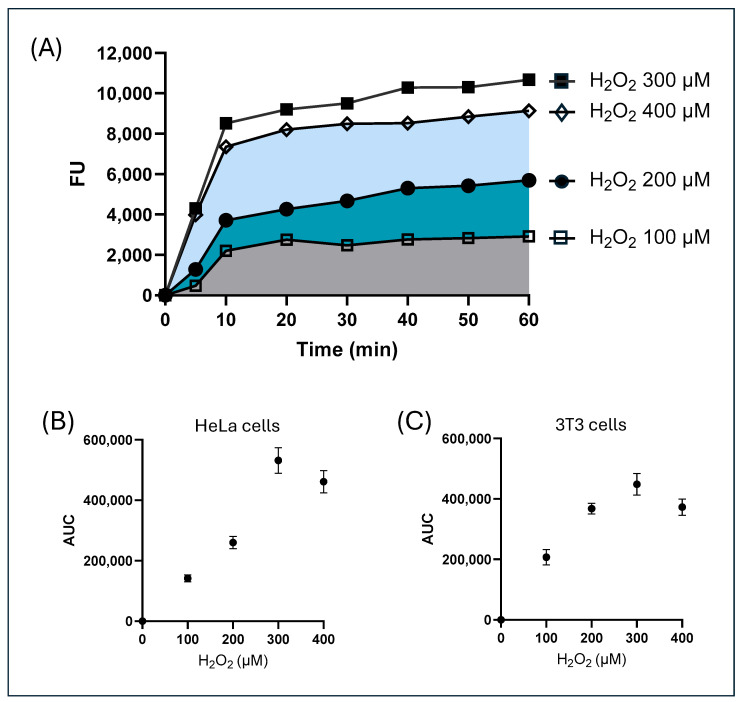
(**A**) The time course of the intracellular fluorescence of HeLa cells charged with the probe CM-H_2_DCFDA and exposed to H_2_O_2_ (100 µM–400 µM). The data were normalized by subtracting, for each time point and each concentration of H_2_O_2_, the values of the fluorescence recorded in non-H_2_O_2_-exposed cells. The graph shows the area under each curve (AUC). **□** = H_2_O_2_ 100 µM, ● = H_2_O_2_ 200 µM, ▪ = H_2_O_2_ 300 µM, and ◊ = H_2_O_2_ 400 µM. The area under curve (AUC) calculated for each curve of HeLa cells (**B**) or 3T3 cells (**C**) was plotted against the corresponding H_2_O_2_ concentration Data are expressed as mean ± SEM of three independent experiments.

In order to preliminarily set up intracellular prooxidant conditions in both HeLa cells and 3T3 cells for application in the Trolox exposure experiments, the cells were exposed to H_2_O_2_ for 1 h at concentrations ranging from 100 µM to 400 µM and then charged with the ROS-sensitive probe CM-H_2_DCFDA. The increase in the fluorescence of the probe was monitored over time. Figure 3A shows a representative time course obtained for HeLa cells; a similar graph was obtained for 3T3 cells. The data were normalized by subtracting, for each time point and each concentration of H_2_O_2_, the values of the fluorescence recorded in non-H_2_O_2_-exposed cells in order to assess the only observed effect caused by the prooxidant conditions induced by hydrogen peroxide. As observed in Figure 3A, the curves showed a hyperbolic increase over time. The time course of the intracellular fluorescence recorded in non-H_2_O_2_-exposed cells compared to H_2_O_2_-exposed cells is reported in Figure S1 (see Supplementary Material).

For each H_2_O_2_ concentration tested, the area under each fluorescence curve (AUC) was calculated (Figure 3A), representing the total amount of reactive species present in the intracellular environment in the observation time. The use of the area under the curve for the kinetic experiments integrates the response–time curve, providing a single value that represents the total effect, regardless of momentary fluctuations, capturing both the intensity and the duration of the response.

The AUC values obtained for each H_2_O_2_ concentration tested were plotted against the concentrations of hydrogen peroxide used either for HeLa cells (Figure 3B) or 3T3 cells (Figure 3B). The resulting curves show a dose-dependent behavior with a linear trend up to the value of 300 µM, while no further increase in the response was observed in both HeLa cells and 3T3 cells at higher H_2_O_2_ concentrations. It is known that CM-H_2_DCFDA is oxidized by H_2_O_2_ in a dose-dependent manner, with fluorescence saturating at high concentrations [31,32]. Therefore, in our experimental design 300 µM hydrogen peroxide was chosen to test the antioxidant properties of Trolox under prooxidant conditions.

### 2.3. Effect of Trolox on Intracellular ROS in HeLa Cells and 3T3 Cells Under H_2_O_2_-Induced Prooxidant Conditions

**Figure 4:**
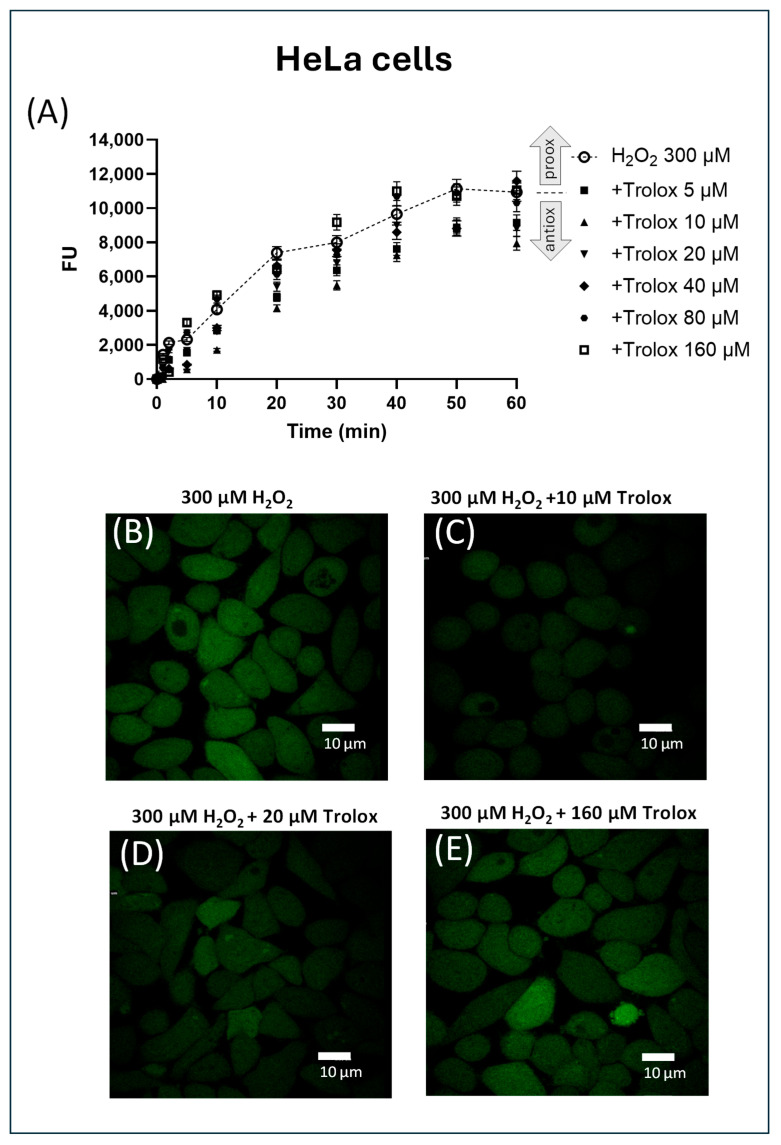
(**A**–**E**) Effect of increasing concentrations of Trolox on H_2_O_2_ (300 µM)-induced fluorescence in CM-H_2_DCFDA-charged HeLa cells. The fluorescence was spectrofluorimetrically recorded over 1 h. The data were normalized by subtracting, for each time point and each concentration of H_2_O_2_, the values of the fluorescence recorded in non-H_2_O_2_-exposed cells. The trace corresponding to the only H_2_O_2_ is indicated by a dotted line. (**B**–**E**) Representative images of HeLa cells pre-exposed to Trolox for 1 h at different concentrations ((**B**) = 0, (**C**) = 10 µM, (**D**) = 20 µM, (**E**) = 160 µM), charged with the probe, and then treated with H_2_O_2_ 300 µM. The cells were visualized after 30 min exposure to H_2_O_2_.

**Figure 5:**
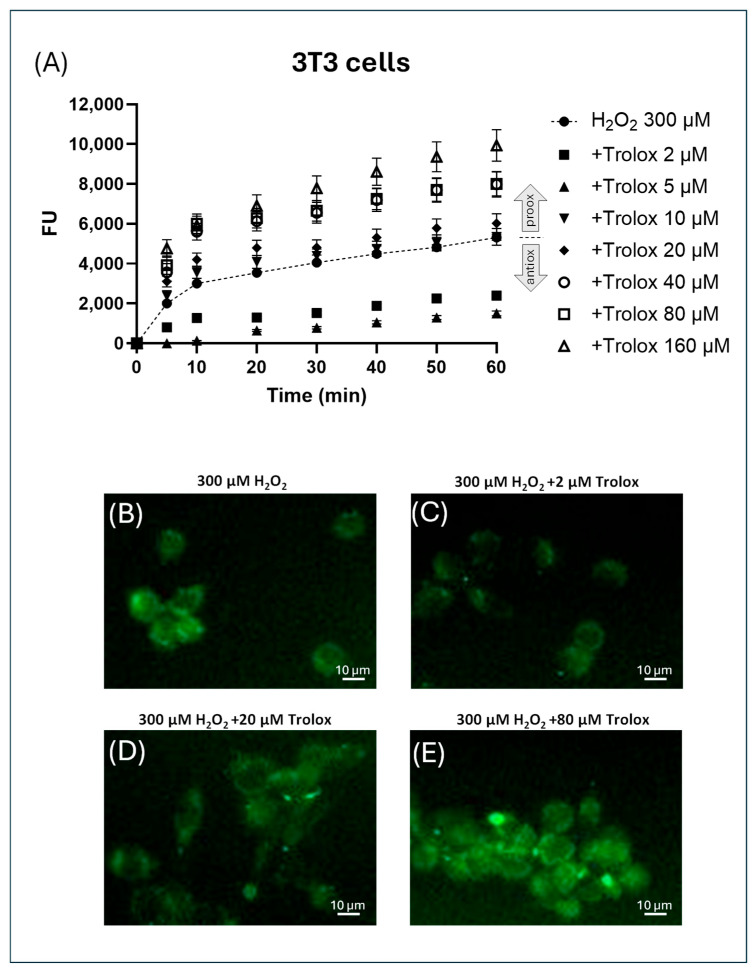
(**A**–**E**) Effect of increasing concentrations of Trolox on H_2_O_2_ (300 µM)-induced fluorescence in CM-H_2_DCFDA-charged 3T3 cells. The fluorescence was spectrofluorimetrically recorded over 1 h. The trace corresponding to the only H_2_O_2_ is indicated by a dotted line. The data were normalized by subtracting, for each time point and each concentration of H_2_O_2_, the values of the fluorescence recorded in non-H_2_O_2_-exposed cells. (**B**–**E**) Representative images of 3T3 cells pre-exposed to Trolox for 1 h at different concentrations ((**B**) = 0, (**C**) = 2 µM, (**D**) = 20 µM, (**E**) = 160 µM), charged with the probe, and then treated with H_2_O_2_ 300 µM. The cells were visualized after 30 min exposure to H_2_O_2_.

When either HeLa cells or 3T3 cells were preincubated with Trolox for 1 h at different concentrations and then exposed to H_2_O_2_ 300 µM (Figure 4A and Figure 5A), they showed the typical hyperbolic increment of their fluorescence over time.

In HeLa cells, none of the fluorescence time courses, except for the one corresponding to cells preincubated with 160 µM Trolox, showed increased fluorescence values compared to the curve with H_2_O_2_ alone (indicated by the dashed line). This suggests general antioxidant behavior at concentrations lower than 160 µM; however, the response does not follow a clear dose-dependent trend. This is also confirmed by confocal microscopy images (Figure 4B–E), since a marked inhibition of peroxide-induced fluorescence was observed in the image of cells exposed to 10 µM Trolox, while a less pronounced inhibition was evident at 20 µM and no inhibition and slight prooxidant behavior at 160 µM. The fluorescence microscopically appeared to be fairly evenly distributed throughout the cytoplasm, suggesting that after de-esterification, CM-H_2_DCFDA localizes to the cytosol in these cells.

In 3T3 cells only the concentrations of 2 and 5 µM Trolox showed decreased fluorescence values compared to the curve with H_2_O_2_ alone (indicated by the dashed line), indicating an antioxidant behavior (Figure 5A). All the other concentrations tested showed increased fluorescence values compared to the curve with H_2_O_2_ alone, expressing a prooxidant behavior (Figure 5A). This was also confirmed by the visualization of the cells by widefield fluorescence microscopy, as reported in the representative images in Figure 5B–E), where a marked inhibition of peroxide-induced fluorescence was observed in the image of cells exposed to 2 µM Trolox, no inhibition at 10 µM, and prooxidant behavior at 80 µM.

**Figure 6:**
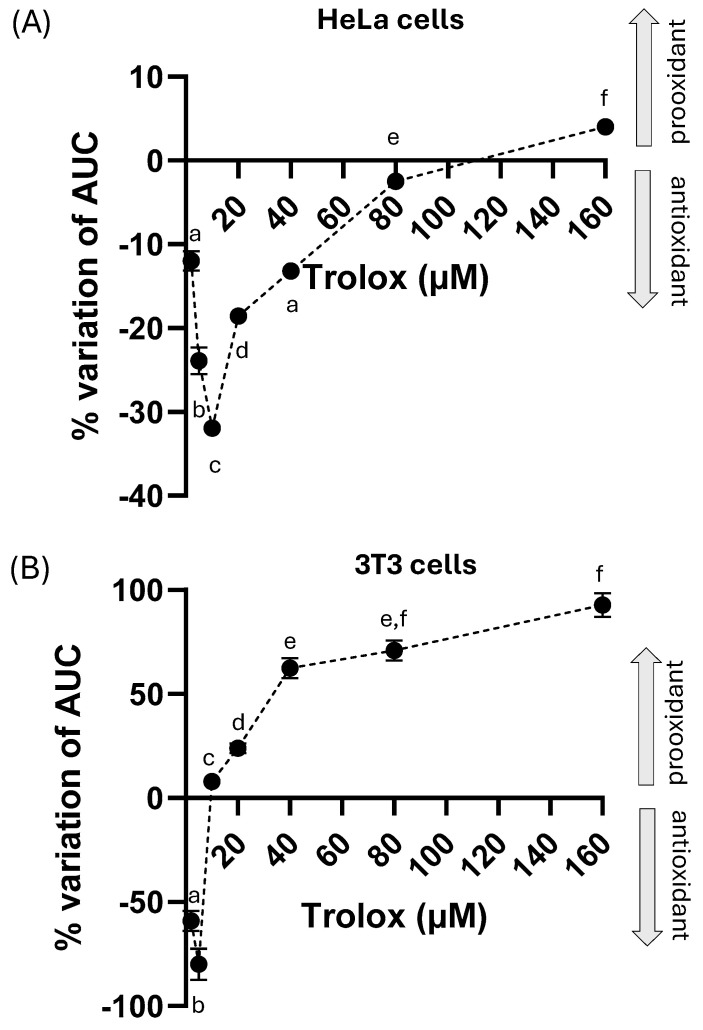
(**A**,**B**) Percentage variation of the effect of Trolox (1 h incubation) at different concentrations (from 2 µM to 160 µM) on the H_2_O_2_ (300 µM)-induced fluorescence of HeLa cells (**A**) and 3T3 cells (**B**) charged with the fluorescent probe CM-H_2_DCFDA. Same details as Figure 2. The statistical significance of the data was analyzed by one-way ANOVA followed by Tukey’s multiple comparison post-test. Different letters indicate statistically significant differences (*p* < 0.05) between concentrations.

The spectrofluorimetric response of both cell types was quantified as the AUC of the time course curves for all the Trolox concentrations used (Figure 6A,B), and the AUC percentage variation was calculated with respect to the time course of only H_2_O_2_. A non-monotonic behavior in the percentage variation was observed for the two cell types (Figure 6A,B). In particular, HeLa cells (Figure 6A) showed a dose-dependent increase in antioxidant activity (expressed as a negative percentage change) at low Trolox concentrations up to 10µM, followed by a reversal of this trend with a progressive dose-dependent decrease in antioxidant activity and disappearance at around 80 µM. At a concentration of 160 µM, a slight prooxidant activity was observed. Therefore, as the concentration of Trolox increases beyond 10 µM, its protective effect against an oxidative challenge appears to decrease until it is nullified, and a prooxidant behavior rises at concentrations above 100 µM. In contrast, 3T3 cells exposed to H_2_O_2_ showed an antioxidant behavior only at the lowest concentrations (2–5 µM), while at 10 µM Trolox already exhibited marked prooxidant effects (Figure 6B).

### 2.4. Comparison with Previous Studies

The results obtained in this study were compared with literature data as reported in Table 1, which summarizes the antioxidant/prooxidant activity of Trolox across different cell models and in different experimental conditions. By comparison with literature data, the results of the present study under baseline conditions (no antioxidant added) confirm the data of Giordano et al. [3] carried out on HeLa cells but using prolonged exposure time (24 h). The present work adds further information, namely, that the prooxidant effect of Trolox under baseline conditions at high concentrations can emerge early, even after relatively short exposures (1 h), and is related to the specific redox environment of the cells. Furthermore, the results of the present study under H_2_O_2_-induced oxidant conditions are consistent with the results obtained on another cellular model, rat hepatocytes [33], where also in this case a biphasic behavior of Trolox was documented, although the type of oxidative stress is different. By comparing the present study with the results of Diaz et al. [34], both studies agree on the fact that Trolox becomes prooxidant at high concentrations (≥100 µM). The threshold seems relatively similar, even if in our study the effect emerges more rapidly, already after 1 h, while in the study by Diaz et al. [34] chronic effects are observed in pro-apoptotic contexts induced by As_2_O_3_, with cell type-dependent outcomes.

## 3. Discussion

The present study provides new insights into the concentration dependence of the dual antioxidant/prooxidant behavior of Trolox in HeLa cells and 3T3 cells, contributing to clarifying its biphasic behavior under short-term (1 h) exposure both under baseline conditions (no oxidant added) and under prooxidant conditions (H_2_O_2_ added). The use of two biologically distinct cellular models, 3T3 fibroblasts and HeLa cells, selected for their complementary redox profiles and physiological relevance, allowed us to better understand the dual antioxidant and prooxidant behavior of Trolox. The use of a short-term exposure experimental approach (1 h) enabled the simulation of acute oxidative insults and expanded previous observations obtained with prolonged exposure (24 h).

Prooxidant conditions were experimentally designed as exposing the cells to 300 µM H_2_O_2_, which closely resembles the concentrations encountered during acute and chronic inflammation in vivo. H_2_O_2_ is a key reactive oxygen species produced by activated immune cells during the inflammatory response [22], and extracellular H_2_O_2_ levels can locally rise under pathological conditions. As demonstrated by several studies, H_2_O_2_ concentrations may reach the low micromolar to sub-millimolar range in inflamed tissues, ischemic regions, and tumor microenvironments [22,26,27,28]. Therefore, the use of 300 µM H_2_O_2_ in our model reproduces conditions similar to biologically realistic oxidative stress, such as that experienced by cells within an inflammatory microenvironment.

The ROS-sensitive intracellular probe used for this study was CM-H_2_DCFDA. The oxidation of this probe is not selective for a single type of reactive oxygen species (ROS) [33] since the probe can be oxidized not only by H_2_O_2_ but by several oxidizing species, including hydroxyl radicals (·OH), reactive species generated from the interaction between H_2_O_2_ and peroxidase or heme, NO_2_ generated via the myeloperoxidase/H_2_O_2_/NO_2_^−^ system, and reactive intermediates from peroxynitrite (ONOO^−^/ONOOH) decomposition. Consequently, it does not provide a direct measurement of intracellular H_2_O_2_ levels, but it measures the general intracellular oxidant levels [30]. The probe has been widely demonstrated to be sensitive enough to reveal both endogenous and exogenous ROS production [29,31,36,37,38,39,40]. In our experimental model, during the H_2_O_2_-induced prooxidant conditions, a hyperbolic increase in fluorescence following H_2_O_2_ exposure was observed, reflecting a broader increase in intracellular oxidative stress conditions induced by H_2_O_2_ rather than a direct, time-dependent rise in intracellular H_2_O_2_ concentration.

The results of this study on the two cell models support the concept that the intracellular redox activity of Trolox is not only concentration-dependent but also modulated by the specific intracellular oxidative environment. Indeed, consistent differences between the two models in either basal or oxidative stress conditions were observed.

Under basal conditions, 3T3 fibroblasts, representative of a non-transformed, low-ROS cellular environment [29], responded to Trolox with a modest but consistent antioxidant behavior across the full concentration range (2–160 µM), tested as assessed by the gradual, monotonic decrease in CM-H_2_DCFDA fluorescence percentage variation. In contrast, HeLa cells, characterized by a prooxidative metabolic state and elevated basal ROS levels due to their cancerous origin [30], exhibited a biphasic response to Trolox. In HeLa cells Trolox exerted antioxidant effects at low concentrations (≤10 µM) but shifted toward a prooxidant profile at higher doses, presumably due to accumulation of phenoxyl radicals [31] and saturation of cellular reducing systems.

To explain the mechanisms underlying the different behaviors observed in the two cell types, we must consider that high intracellular ROS concentrations can promote the oxidation of Trolox into phenoxyl radicals—especially under conditions of elevated oxidative stress. It is known that Trolox undergoes rapid one-electron oxidation by various ROS [9,41,42,43] and that enzymatic mechanisms (e.g., peroxidases, lipoxygenases) can oxidize Trolox to its phenoxyl radical form [41,42,44]. As a result, the formation of Trolox phenoxyl radicals becomes more likely when ROS levels are elevated, as occurs under oxidative stress [41,45]. Therefore, considering the distinct redox conditions of the two cell types, it is reasonable to suggest that Trolox could undergo more pronounced oxidation in HeLa cells than in 3T3 cells under basal conditions, potentially resulting in different antioxidant/prooxidant behavior. In HeLa cells, at concentrations up to 10 µM, Trolox can effectively scavenge intracellular ROS. In this condition intracellular antioxidants such as glutathione and ascorbic acid [9] are not overwhelmed and can regenerate Trolox from its phenoxyl radical form. However, at concentrations exceeding 20 µM, the balance could shift toward a prooxidant effect. This transition could be due to the accumulation of Trolox-derived phenoxyl radicals that, in the absence of sufficient reducing agents such as glutathione or ascorbate, are not efficiently recycled. In 3T3 cells the formation of Trolox phenoxyl radicals can be either minimal or effectively controlled. The favorable redox status of 3T3 cells, characterized by low endogenous ROS production and a robust pool of intracellular reductants such as glutathione, NADPH, and ascorbate [46], likely plays a central role in the antioxidant behavior observed. Conversely, under H_2_O_2_-induced oxidative stress, in HeLa cells low concentrations of Trolox (≤10 µM) retained antioxidant activity. However, this protective activity declined progressively with increasing concentrations and was lost at 160 µM, where prooxidant effects become apparent. In 3T3 cells, only the lowest concentrations of Trolox (2–5 µM) showed antioxidant activity in the presence of H_2_O_2_. From 10 µM onward, Trolox induced greater oxidative stress than H_2_O_2_ alone, demonstrating a pronounced and early shift toward prooxidant activity in this model.

Also in this case, the different responses between the two cell types could reflect differences in their redox status. It is known that in HeLa cells exposure to H_2_O_2_ activates antioxidant systems, particularly the Trx system, to maintain redox homeostasis [47]. Conversely, there is no direct evidence in the literature that H_2_O_2_ activates antioxidant enzymes in 3T3 cells. Therefore, to explain the Trolox behavior in the two cell types under H_2_O_2_ exposure, it can be suggested that HeLa cells possess an already-H_2_O_2_-triggered antioxidant network, which enables them to transiently buffer the added oxidative insult coming from Trolox-derived phenoxyl radicals. At low Trolox concentrations, the compound synergizes with this system, enhancing the detoxification of H_2_O_2_. However, at higher concentrations, accumulation of Trolox phenoxyl radicals in an already-strained system leads to a dose-dependent reduction in the antioxidant behavior and reversal to prooxidant behavior at 160 µM Trolox. In contrast, 3T3 cells under H_2_O_2_ stress display limited adaptability. Although they manage Trolox well in basal conditions, the acute oxidative challenge overwhelms their uninduced antioxidant machinery. The co-presence of H_2_O_2_ and high-dose Trolox accelerates the oxidation of Trolox into phenoxyl radicals, which in this redox-compromised state cannot be efficiently reduced. This leads to an early onset of prooxidant effects, observed even at intermediate Trolox concentrations.

Overall, this is one of the few studies to compare side-by-side a cancer cell line (HeLa) with high basal ROS [30], and a non-transformed fibroblast line (3T3) with low ROS [29], providing new insights into how cellular redox status modulates Trolox effects. The study demonstrated that the same Trolox concentration can have different effects depending on both cell type and oxidative condition. In comparison with the existing literature, the findings of the present study extend our previous work [3], which investigated Trolox in HeLa cells but under baseline conditions and with longer exposure. Moreover, with reference to the work of the authors of [33], who used rat hepatocytes exposed to cumene hydroperoxide or a peroxidase/H_2_O_2_ system, further supports the context- and concentration-dependent behavior of Trolox. Their study found that concentrations below 20 µM protected against lipid peroxidation and cytotoxicity, whereas concentrations around 100 µM led to increased malondialdehyde (MDA) formation, glutathione (GSH) oxidation, and cell damage, confirming a prooxidant effect at higher doses. While the cell model and oxidant differ (cell line in our study, primary cell in the Tafazoli study), both studies highlight the threshold behavior of Trolox: protective at low concentrations, harmful at high doses. In a different experimental context, Díaz et al. [34] investigated the effects of 100 µM Trolox in several hematopoietic cell lines and murine fibroblasts under chronic oxidative stress induced by arsenic trioxide (As_2_O_3_). They observed prooxidant effects, including oxidative stress and apoptosis in NB4, AsR2, and IM9 cells, but a protective antioxidant effect in fibroblasts, presumably due to different redox intracellular environments and antioxidant capacities. Notably, their exposure times ranged from 3 to 6 days, indicating long-term effects. Our study complements these findings by showing that prooxidant activity can also emerge rapidly and that the redox effects of Trolox are influenced not only by concentration and cell type but also by the nature (acute vs. chronic) and mechanism of oxidative challenge. Moreover, they broaden the current understanding of the prooxidant activity of Trolox, which in previous literature had been primarily characterized by the presence of metals [16,18,34,42].

While earlier studies have predominantly described Trolox prooxidant behavior in the context of transition metal-catalyzed redox reactions [16,18,34,42], the current study demonstrates that phenoxyl radical-mediated prooxidant activity can occur in the absence of metal catalysts, further supporting the idea that Trolox’s redox behavior is highly context- and concentration-dependent.

Since Trolox is a water-soluble analog of α-tocopherol, it is important to contextualize its observed dual antioxidant/prooxidant behavior by comparing it with that of α-tocopherol, the natural lipophilic form of vitamin E. Both compounds share the redox-active chromanol ring, which is central to their antioxidant activity through hydrogen atom donation and radical scavenging [48]. Like Trolox, α-tocopherol exhibits well-documented antioxidant properties in biological membranes, where it protects polyunsaturated fatty acids from lipid peroxidation [48]. However, α-tocopherol can also exert prooxidant effects under certain conditions, such as high concentrations, presence of transition metals, and/or low levels of co-antioxidants [49,50,51,52,53,54]. Bowry and colleagues [54] notably demonstrated that in low-density lipoproteins (LDL), α-tocopherol can become a prooxidant, particularly when co-antioxidants are depleted; it generates tocopheroxyl radicals that react with polyunsaturated fatty acids in LDL, thus propagating lipid peroxidation chain reactions. Comparing the prooxidant effects reported for α-tocopherol and the effects observed in the present study for Trolox, it is plausible that phenoxyl radical formation underlies the prooxidant effects of both Trolox and α-tocopherol, given their shared redox-active chromanol moiety. However, due to its lipophilic nature, α-tocopherol primarily accumulates in cellular membranes, whereas Trolox distributes in the aqueous cytosolic compartment. This distinction may affect the site and extent of their effects, as well as the efficiency of phenoxyl radical recycling, which is highly compartment-dependent [55].

Overall, our findings, although based on the use of a single fluorescent probe (CM-H_2_DCFDA), reflect a broader principle applicable to chromanol-based antioxidants, including α-tocopherol: namely, that dose, cellular redox state, and antioxidant regeneration capacity collectively determine the antioxidant or prooxidant behavior of the compound. Future studies incorporating complementary approaches will be essential to deepening the mechanistic understanding of Trolox’s dual redox behavior and to clarifying how its activity can be modulated by the intracellular antioxidant system.

## 4. Methods

### 4.1. Materials

All chemicals were reagent-grade. Cell culture materials were acquired from EuroClone (Paignton-Devon, UK). The cell-permeant probe 5-(and-6)-chloromethyl-20,70-dichlorodihydrofluorescein diacetate (CM-H_2_DCFDA) was purchased from Life Technologies-Molecular Probes (Waltham, MA, USA). All the other reagents were purchased from Merck (Darmstadt, Germany). Trolox was used as a commercially available racemic mixture.

HeLa cells were purchased from ATCC (Manassas, VA, USA).

### 4.2. Assessment of Trolox Intracellular Antioxidant/Prooxidant Activity in HeLa Cells and 3T3 Cells

The intracellular antioxidant/prooxidant activity of Trolox was assessed in HeLa cells and 3T3 cells charged with the ROS-sensitive probe, 5-(and-6-)-chloromethyl-20,70-dichlorodihydrofluorescein diacetate, and acetyl ester (CM-H_2_DCFDA) (Thermo Fisher Scientific, Waltham, MA, USA) according to [3,31].

HeLa cells and 3T3 cells were cultured as a monolayer in Dulbecco’s Modified Eagle’s Medium (DMEM) containing 4500 mg/L glucose, supplemented with 10% fetal bovine serum (FBS), 40 IU/mL penicillin G, 2 mM L-glutamine, and 100 µg/mL streptomycin, under a humidified atmosphere of 95% air and 5% CO_2_ at 37 °C.

Cells were seeded at a density of 1 × 10^4^ cells/mL into Corning^®^ 96-well black, flat-bottom, polystyrene, tissue culture-treated microplates and incubated for 24 h to allow for cell adhesion.

For the assessment of the intracellular effect of Trolox under baseline conditions, HeLa cells and 3T3 cells were first incubated with increasing concentrations of Trolox (ranging from 2 to 160 µM) for 1 h. Then, they were washed three times, charged with the cell-permeant fluorescent probe CM-H_2_DCFDA (5-(and-6)-chloromethyl-2′,7′-dichlorodihydrofluorescein diacetate) at 5 µM (30 min incubation), and then washed three times. CM-H_2_DCFDA is a cell-permeant, non-fluorescent probe that diffuses into cells, where intracellular esterases cleave the diacetate groups, trapping the probe inside. Upon oxidation by reactive oxygen species (ROS), it is converted to the highly fluorescent 2′,7′-dichlorofluorescein (DCF), allowing for detection of ROS activity within living cells (30). Fluorescence was measured using a Cytation 5 multi-mode microplate reader (BioTek Instruments, Inc., Winooski, VT, USA) with excitation/emission wavelengths of 492–495 nm and 517–527 nm, respectively.

For the assessment of the intracellular effect of Trolox under H_2_O_2_ challenges, HeLa cells and 3T3 cells were first incubated with Trolox at different concentrations (ranging from 2 to 160 µM) for 1 h. Then, they were washed three times, charged with the cell-permeant fluorescent probe CM-H_2_DCFDA 5 µM for 30 min, and then washed three times again. Afterwards, the cells were exposed to H_2_O_2_ (ranging from 100 to 400 µM) for 1 h. The time course of the CM-H_2_DCFDA fluorescence variation under H_2_O_2_ exposure was recorded as reported above.

### 4.3. Visualization of HeLa Cells by Confocal Microscopy

HeLa cells were plated at a density of 1 × 10^4^ cells per mL into a chambered coverslip (IBIDI, Gräfelfing, Germany), incubated for 24 h for the adhesion of the cells to the bottom of the plate, and then charged with CM-H_2_DCFDA (as reported above). The cells were viewed under different experimental conditions using an A1 NIKON confocal laser scanning unit coupled with a NIKON Ti microscope. Cells were visualized by a Plan Apo 60x 1.40 Oil objective (Nikon, Tokyo, Japan). The 488 nm laser line was used. Each measurement was performed in at least five randomically chosen fields. Unlabeled cells did not exhibit any detectable fluorescence under the conditions used. Images were acquired and analyzed by Nis-Elements AR NIKON software (version 5.42.06).

### 4.4. Visualization of 3T3 Cells by Widefield Fluorescence Microscopy

T3 cells were plated at a density of 1 × 10^4^ cells per mL into a Corning^®^ 96-well black, flat-bottom, polystyrene, tissue culture-treated microplate and incubated for 24 h to allow for cell adhesion. Then, the cells were charged with CM-H_2_DCFDA (as reported above) and were viewed under different experimental conditions using the widefield fluorescence read mode of a Cytation 5 multi-mode microplate reader (BioTek Instruments, Inc., Winooski, VT, USA) provided with an GFP/FITC/Alexa Flour 488—EX: 469/35 EM:525/39 filter set.

### 4.5. Statistical Analysis

Data are presented as mean ± S.E.M. For multiple comparisons, analysis was performed by one-way ANOVA followed by Tukey’s multiple comparison post-test, as specified in the captions of figures. The GraphPad Prism (version 10) software was used for all the analyses and graphing.

## 5. Conclusions

In conclusion, this study provides new insights into the dual redox behavior of Trolox by demonstrating that both its antioxidant and prooxidant effects can manifest within a short exposure period (1 h) and that they are highly dependent on cell type and oxidative context. Through the comparative analysis of the responses observed in the two cell models, the study demonstrated that the same concentrations of Trolox can produce varying effects depending on the cell type and the oxidative intracellular environment, offering a more subtle understanding of the multifaceted aspects of antioxidant supplementation. These results emphasize that antioxidant supplementation cannot be considered always beneficial and must be contextualized based on dose, exposure time, and cellular redox characteristics. Based on our findings, several directions emerge for future research, including mechanistic studies on phenoxyl radical formation and fate and exploration of redox adaptability across cell types for the selection of antioxidant treatments based on cellular redox phenotypes.

## Figures and Tables

**Table 1:** Antioxidant/prooxidant activity of Trolox across different cell models and in different experimental conditions.

Cell Type	Trolox Concentration	Observed Effects	Pro/Antioxidant	Conditions	Exposure Duration	Ref
HeLa cells	2–20 µM	Non-monotonic decrease in intracellular ROS	Antioxidant (non-monotonic behavior)	Baseline (no added oxidant)	1 h	Present study
HeLa cells	>20 µM	Increased ROS	Prooxidant	Baseline (no added oxidant)	1 h	Present study
HeLa cells	>10–80 µM	Non-monotonic decrease in intracellular ROS	Antioxidant (non-monotonic behavior)	H_2_O_2_	1 h	Present study
HeLa cells	160 µM	Increased ROS	Prooxidant	H_2_O_2_	1 h	Present study
HeLa cells	2.5–15 µM	Decreased intracellular ROS	Antioxidant	Baseline (no added oxidant)	24 h	[3]
3T3 cells	2–160 µM	Slight monotonic decrease in intracellular ROS	Antioxidant (monotonic behavior)	Baseline (no added oxidant)	1 h	Present study
3T3 cells	2–5 µM	Non-monotonic decrease in intracellular ROS	Antioxidant (non-monotonic behavior)	H_2_O_2_	1 h	Present study
3T3 cells	10–160 µM	Non-monotonic increase in intracellular ROS	Prooxidant (non-monotonic behavior)	H_2_O_2_	1 h	Present study
HeLa cells	>20 µM	Increased intracellular ROS, apoptosis	Prooxidant	Baseline (no added oxidant)	24 h	[3]
Rat hepatocytes	<20 µM	Inhibited cumene hydroperoxide-induced LPO and cytotoxicity	Antioxidant	90µM Cumene peroxide	2 h	[33]
Rat hepatocytes	100 µM	LPO (MDA formation); GSH oxidation; cytotoxicity	Prooxidant	peroxidase/H_2_O_2_	2 h	[33]
Rat astrocytes	250 µM	Depolarization of mitochondria	Prooxidant	CuSO_4_	15 min	[35]
NB4 cells	100 µM	Oxidative stress; apoptosis	Prooxidant	As_2_O_3_	6 days	[34]
AsR2 cells	100 µM	Oxidative stress; apoptosis	Prooxidant	As_2_O_3_	6 days	[34]
IM9 cells	100 µM	Oxidative stress; apoptosis	Prooxidant	As_2_O_3_	6 days	[34]
Mouse fibroblasts	100 µM	Decreased As_2_O_3_^−^ mediated apoptosis	Antioxidant	As_2_O_3_	3 days	[34]
Erythrocyte membrane	50 µM	Lipid oxidation	Prooxidant	Fe^3+^	30 min	[16]
Cell-free system	5 mM	Hydroxyl radical formation	Prooxidant	Cr(VI), H_2_O_2_	30 min	[18]

## Data Availability

The raw data supporting the conclusions of this article will be made available by the authors on request.

## Supplementary Materials

The following supporting information can be downloaded at: https://www.mdpi.com/article/10.3390/molecules30183755/s1, Figure S1: Basal fluorescence of HeLa cells expressed as a percentage of that in 3T3 cells; Figure S2: Time course of the intracellular fluorescence of HeLa cells charged with the probe CM-H_2_DCFDA.
