# Supplementary material for: Intracellular Dual Behavior of Trolox in HeLa Cells and 3T3 Fibroblasts Under Basal and H2O2-Induced Oxidative Stress Conditions
Source: Molecules. 2025 Sep 16;30(18):3755. doi: 10.3390/molecules30183755 (PMC12472327; doi:10.3390/molecules30183755)

Figure S1. Basal fluorescence of HeLa cells expressed as a percentage of that in 3T3 cells. Data are presented as the mean ± SEM. The statistical significance of data was analyzed by Student's t-test. **= P<0.01.

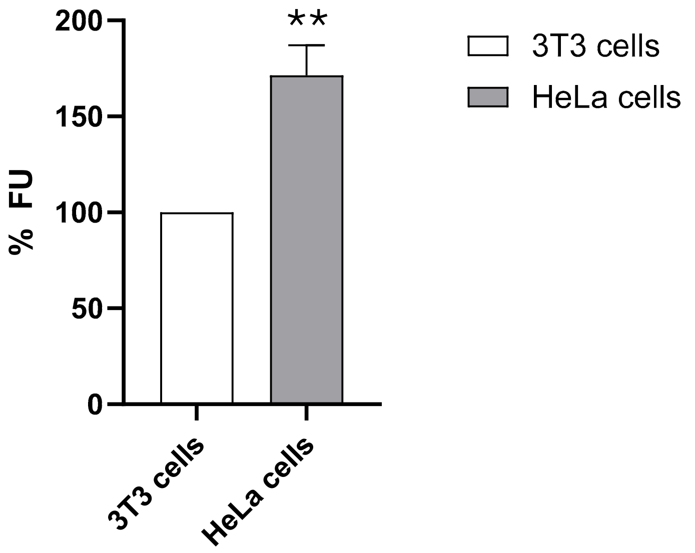

Figure S2: Time-course of the intracellular fluorescence of HeLa cells charged with the probe CM-H_2DCFDA in the presence (□) and in the absence (●) of H_2O_2 (300 μM μM). The data were normalized by subtracting, for each time point and each concentration of H_2O_2, the values of the fluorescence recorded at time 0.

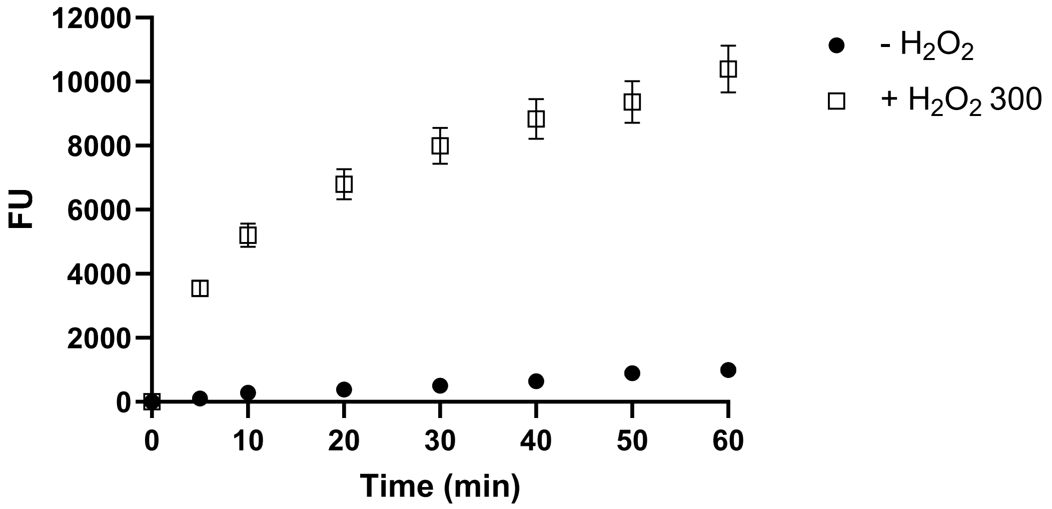

Supplement: Supplementary file 1 [file molecules-30-03755-s001.zip › molecules-3725281-supplementary.pdf]
